# Treatment for Wear and Osteolysis in Well-Fixed Uncemented TKR

**DOI:** 10.1155/2013/398298

**Published:** 2013-02-11

**Authors:** Leah Nunez, Brandon Broome, Tom Pace, Melinda Harman

**Affiliations:** ^1^Department of Bioengineering, Clemson University, Clemson, SC 29634, USA; ^2^Steadman Hawkins Clinic of the Carolinas, Greenville, SC 29615, USA; ^3^Department of Orthopaedics, USCSOM-Greenville, Greenville, SC 29208, USA

## Abstract

*Background.* Traditionally, osteolysis around total knee replacements (TKRs) is treated with complete revision. In certain subsets, polyethylene insert exchange and bone grafting may be applicable. This study reports the clinical outcomes for selective bone grafting in patients with osteolysis without complete revision of the TKR. *Methods.* This retrospective study analyzes 10 TKRs (9 patients, 66.5 ± 6.1 years old) presenting with osteolysis and revised after 8.7 ± 1.9 years of in vivo function. At index TKR, all patients were implanted with uncemented prosthesis and modular polyethylene insert with anteroposterior articular constraint (Ultracongruent, Natural Knee II, Sulzer Medica). The surgical technique for treating the osteolysis included removal of necrotic bone tissue using curettage, filling of the defect with bone graft materials, and polyethylene insert exchange. *Results.* Patients have not exhibited any further complications associated with osteolysis after 5.1 ± 2.4 years of followup. Routine radiographic exams show total incorporation of the graft material into the previously lytic regions in all patients. *Conclusion.* In some TKRs with osteolysis and firmly fixed components, the removal of lytic tissue and subsequent defect filling with bone graft materials can be a viable solution. This case series shows complete resolution of osteolysis in all patients with no complications.

## 1. Introduction

Periprosthetic osteolysis is a known complication after cementless total knee replacement (TKR), including cases in which the implant is well fixed and properly aligned [1–9]. A viable treatment option for progressive periprosthetic osteolysis observed after total hip replacement (THR) is polyethylene liner exchange and bone grafting of the osteolytic lesions [10]. Using this treatment method as a model, a polyethylene insert exchange and bone grafting technique was developed to treat patients with progressive periprosthetic osteolysis in cementless TKR. Due to the decrease in survivorship associated with complete TKR revision [11], combined with the increasingly younger patients undergoing TKR, this method may be a viable option for a select group of TKR patients with osteolysis.

Osteolysis is a well-recognized complication after THR that presents diagnostic and treatment challenges [10]. Among patients showing polyethylene wear and acetabular osteolysis who are otherwise asymptomatic for pain without visible cup loosening or malalignment, treatment options include isolated liner exchange or revision of the liner and cup, both in combination with retroacetabular bone grafting [12]. The conditions that qualify a patient for isolated liner exchange are controversial, and as a result, there is debate over the use of this treatment method [10, 13]. Studies have shown that isolated liner exchange has neutral-to-favorable outcomes when compared to revision THR of the liner and cup, with infrequent minor complications and an absence of osteolysis progression [10, 12, 13].

Similar to THR, periprosthetic osteolysis associated with polyethylene wear can occur adjacent to the metal components of TKR. The traditional course of treatment is complete TKR revision [3], but bone grafting and isolated insert exchange may be an option for some osteolytic patients, given the lessons learned from THR. However, isolated insert exchange after TKR has had variable success, suggesting that clear indications and surgical decision models are needed. Rerevision rates of 16% to 25% have been reported at less than five-year followup after isolated insert exchange for instability, wear, and osteolysis in TKR [14, 15]. In contrast, excellent results have been reported for treating focal osteolysis with bone grafting and isolated insert exchange, with rerevision necessary in less than 5% of cases and no evidence of component loosening [16].

The purpose of this study is to systematically assess patients who presented with progressive periprosthetic osteolysis adjacent to well-fixed and well-aligned uncemented TKR and were treated with bone grafting and isolated insert exchange. We define the preoperative and intraoperative surgical decision models used in the clinical evaluation and surgical treatment of these patients and present a retrospective review of outcomes at 1 to 10 years of followup.

## 2. Materials and Methods

We retrospectively reviewed 9 patients (10 cases) who presented with osteolysis adjacent to well-fixed and well-aligned uncemented TKR and were treated with bone grafting and isolated exchange of the tibial polyethylene insert and retention of the femoral and tibial components. The senior surgeon (TP) performed all index TKR between December 1996 and January 2003 and all subsequent bone grafting and isolated insert exchanges between December 2002 and December 2011. Approval for clinical records review was obtained from our Institutional Review Board.

At index TKR, all patients presented with an underlying diagnosis of osteoarthritis. Surgical technique included a subvastus approach with resection of the posterior cruciate ligament, a tibial cut aligned parallel with the posterior slope of the articular surface, and the patella left unresurfaced. All knees were implanted with an uncemented TKR prosthesis (Natural Knee II with Ultracongruent insert, Sulzer Medica, Austin, TX, USA). Femoral and tibial component fixation was enhanced by spreading the cut bone surfaces with a bone slurry reamed from the cancellous bone of the tibial wafer [17, 18], with five tibial baseplates further augmented with insertion of cancellous screws. All patients were followed during routine annual clinical evaluations, including radiographic and physical exams. Knee Society Scores preceding bone grafting and isolated insert exchange for these patients averaged 96.4 ± 5.3.

**Figure 1:**
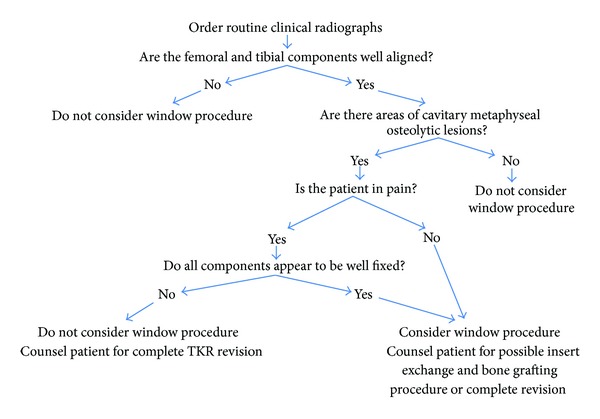
Preoperative surgical decision model.

**Figure 2:**
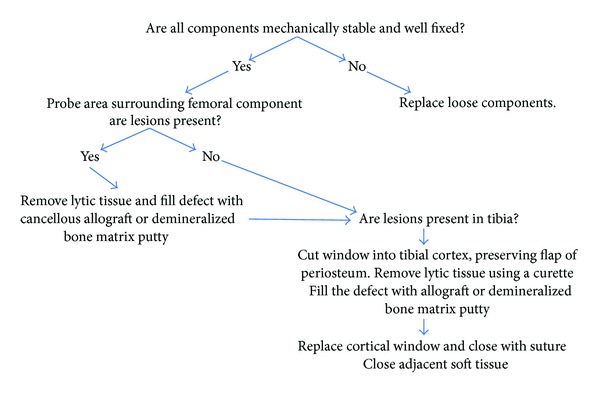
Intraoperative surgical decision model.

The main indication for subsequent surgery, including bone grafting and isolated insert exchange, was periprosthetic osteolysis observed on routine clinical radiographs. All patients were counseled for possible complete revision of all components and the risks associated with the insert exchange and bone grafting procedure were discussed in depth. The preoperative surgical decision for bone grafting and isolated insert exchange, rather than complete revision, was indicated in patients presenting with osteolysis with well-aligned components that appeared well-fixed on pre-operative clinical radiographs (Figure 1). If the osteolytic defect is significant enough to potentially threaten mechanical stability, or a small lesion that increases in size in six months to a year of followup, then the window procedure should be considered as a treatment option. If the lesion disrupts the cortical bone, then the window procedure should not be considered as a treatment option. The maximum lesion size that was operated on in our study was 5.5 cm × 6.0 cm, which we defined as a large lesion. The intra-operative surgical decision to proceed with bone grafting and isolated insert exchange was indicated after the senior surgeon (TP) manually confirmed the joint stability and fixation of all components and confirmed localization of the osteolytic regions (Figure 2). At the time of reoperation, the surgical instruments necessary for a complete revision were available in the event that the metal components were not firmly fixed.

The surgical technique for bone grafting and isolated insert exchange followed an uniform intra-operative surgical decision model (Figure 2). Upon opening the joint, stability and fixation of the femoral and tibial components were manually verified by attempting to remove the femoral and tibial components with the extraction instruments. The polyethylene tibial inserts were removed and visually inspected, noting no gross evidence of delamination on the articular surfaces and scratches and deformation into recessed features on the backside surface. Surgical instruments were used to probe along the bone interface of the femoral component to detect any osteolytic regions. If the regions were discovered, then the cystic area was curetted and bone graft materials were used to fill the defect. The tibial cystic area was then addressed by making a 1 cm by 1 cm window medial to the tibial anterior crest, curettage of the tibial osteolytic lesion, and subsequently packing the defect with bone graft material. The window was then replaced on the proximal tibia and secured with sutures in the overlying soft tissues. A new nonultracongruent polyethylene insert (Sulzer Medica) was snapped onto the existing tibial baseplate, with selection of a less congruent bearing surface in all but the first case. All knees retained their initial PE insert size and thickness, except one knee presenting with excessive pre-operative tightness in which the insert was downsized from 11 mm thickness to 9 mm thickness to allow for better motion. The bone graft material utilized included cancellous allograft, demineralized bone matrix putty, or a combination of the two. The decision for which material to use was dependent on availability at time of surgery.

**Figure 3:**
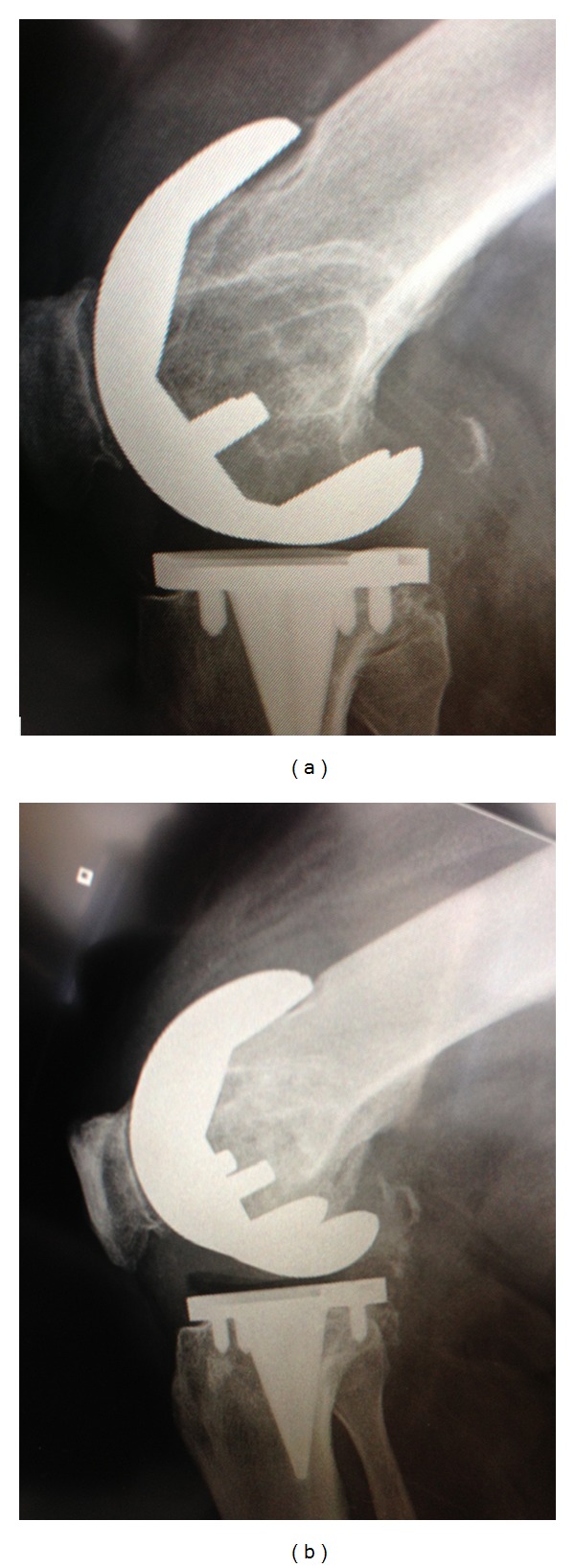
Radiographs of a 67-year-old male who underwent bone grafting and isolated insert exchange for femoral osteolytic region. (a) The prerevision radiograph. (b) Three-month postrevision radiograph.

**Figure 4:**
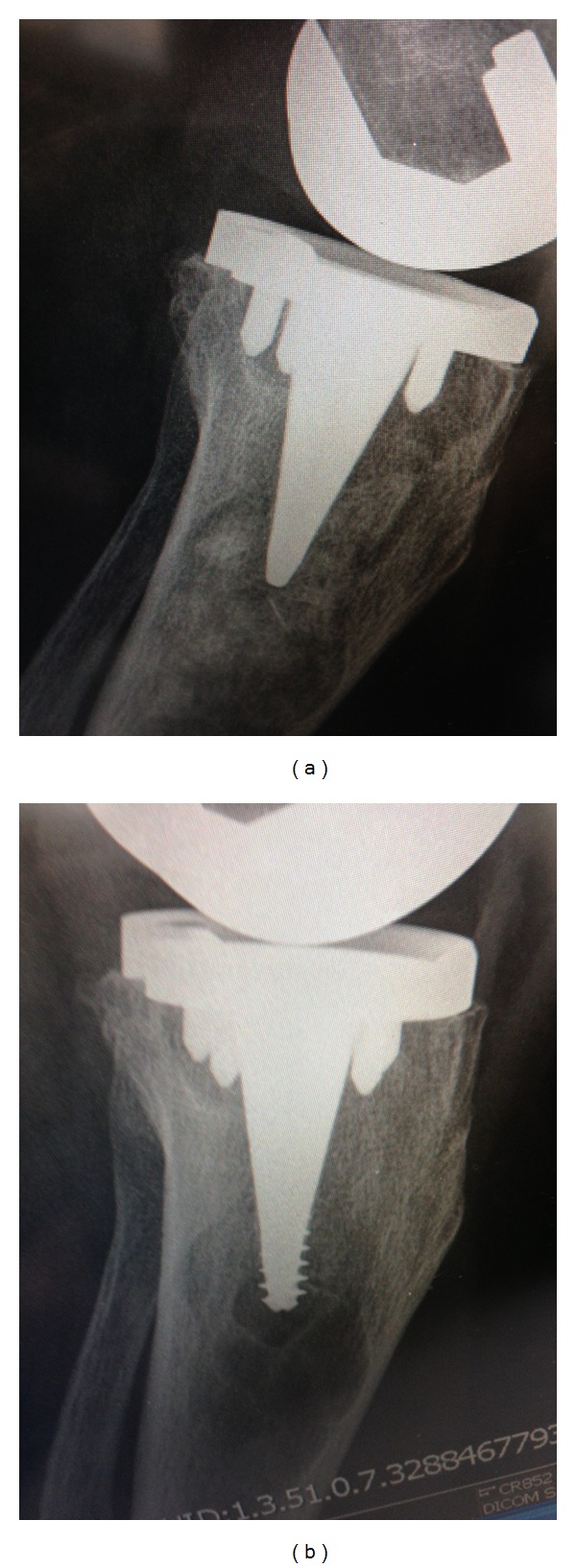
Radiographs for a 64-year-old female patient who underwent bone grafting and isolated insert exchange. (a) prerevision AP view radiograph showing osteolytic region. (b) Three-month postrevision AP view radiograph.

At last followup, clinical outcomes were assessed according to Knee Society Guidelines [19] and radiographs taken before and after the bone grafting and isolated insert exchange procedure were reviewed (Figures 3 and 4). On prerevision radiographs, taken in both the frontal and sagittal planes, radiolucent lines were assessed and osteolytic lesions were classified according to their largest dimension measured on the radiographs. Osteolytic lesions were classified as small if the dimension was less than 2 cm, medium if between 2 cm to 4 cm, and large if greater than 4 cm. On postoperative films, radiolucent lines and the extent of defect healing and graft incorporation were assessed by a fellowship trained arthroplasty surgeon not involved with the index or revision surgery (BB).

## 3. Results and Discussion

### 3.1. Results

There were seven male patients and two female patients treated with bone grafting and isolated insert exchange, including one patient with bilateral procedures completed 4.6 years apart. Patient age averaged 58.2 ± 5.9 (range, 51 to 70) years at the time of index TKR and 66.5 ± 6.1 (range, 58 to 80) years at the time of bone grafting and isolated insert exchange. Body mass index (BMI) averaged 35.6 ± 3.7 (range, 29.6 to 39.1) kg/m^2^. The duration of function for the index TKR averaged 8.7 ± 1.9 (range, 5.7 to 11.4) years prior to the bone grafting and isolated insert exchange procedure, and the length of follow-up time after the procedure averaged 5.1  ±  2.4 (range, 1.0 to 10.0) years. Eight patients were treated with cancellous allograft, two with demineralized bone matrix putty, and one with a mixture of both cancellous allograft and demineralized bone matrix putty.

Clinical followup of these 10 cases revealed no further complications in 100% of the patients, with no reported clinical symptoms of pain and no new areas of osteolysis noted on follow-up radiographs. None of the knees have required additional surgical intervention. One patient suffered multiple long bone fractures including a periprosthetic femoral fracture 2 years later due to a motorcycle trauma but the index TKR components remained intact without a need for revision. The average Knee Society Score improved from 96.4 ± 5.3 (range, 85 to 100) before the bone grafting and isolated insert exchange to 98.5 ± 2.4 (range, 95 to 100) at the most recent followup.

Detailed review of the radiographs revealed findings consistent with the criteria defined in the pre-operative surgical decision model (Figure 1), confirming that no TKRs exhibited radiolucent lines at the interface of the femoral or tibial component prior to bone grafting and isolated insert exchange. Tibial osteolytic lesions assessed on the pre-operative films were graded as small in 2 TKRs, medium in 4 TKRs and large in 3 TKRs. Similarly, femoral osteolytic lesions were graded as medium in 2 TKRs, large in 4 TKRs, and absent in 4 TKRs (Figures 3 and 4). Postoperative radiographs revealed complete graft incorporation into the regions that were previously osteolytic, with an absence of radiolucent lines and no signs of component migration or loosening (Figures 3 and 4).

### 3.2. Discussion

In cementless total joint replacement, periprosthetic osteolysis associated with polyethylene wear is a known complication [1–9]. Isolated exchange of polyethylene bearings in THR and TKR has been used with some success. Due to the more variable outcomes in TKR, we developed uniform pre- and intraoperative surgical decision models to guide our selection of clinical treatment options. The criteria in the surgical decision models provided consistent outcomes at an average of 5 years of followup, with no additional surgical intervention required in these carefully selected patients.

Bone grafting proved useful for treating osteolytic lesions adjacent to both femoral and tibial components, with full graft incorporation effectively eliminating the lesion site and preventing recurrence at 1 to 10 years of followup. These results are more favorable than those of previous studies. Whiteside and Katerberg [20] performed isolated insert exchanges on 49 TKRs for wear with a 6% failure rate at 3 years. In 56 TKR patients presenting with instability or polyethylene wear who were treated with isolated insert exchange, Babis et al. [14] reported a 25% rerevision rate at a mean of three-year followup. Engh et al. [21] performed isolated insert exchange due to wear on 48 TKRs with 7 exchanges failing. Using isolated insert exchange and either bone grafting or cement augmentation to treat 76 TKR patients with polyethylene wear and osteolysis, Griffin et al. [15] reported a 16.2% failure rate after a mean forty-four months. Using a surgical technique similar to the current study, Callaghan et al. [16] reported a 4% rerevision rate in 22 patients at an average of 61-month followup. These variable results can be partially attributed to varied inclusion criteria, especially related to joint instability [14, 15]. Based on previous surgical outcomes combined with our results, the selected use of bone grafting and isolated insert exchange to treat periprosthetic osteolysis appears warranted.

This study utilizes a historical control group for comparison, which is an appropriate comparison for this study because had the femoral and tibial components been removed the residual defect would have required revision-stemmed implants of metal augments, structural bulk allografts, and cancellous allografts options. This control group includes patients who required a revision surgery in which auto- or allograft bone grafts (structural, bulk, or morselized), metal wedges, and modular components were used [22–26]. Peters et al. [25] reported a survivorship of  75%  ±  25% at 99 months of 57 revision TKR after the bone defects were excavated and treated. Cortical allograft bone was used to treat large segmental defects, while cavitary defects were filled with cancellous allograft or autograft bone [25]. Management of bone deficiency with bulk allograft had a reported survivorship of 79.4% to 83% at 8 years of followup [22, 23]. An 85% survivorship was reported at an average of 4.2 years of followup [26]. Mow and Wiedel [24] reported an 84% survivorship for a study of 13 revisions using structural allografts. The decrease in survivorship of revision TKR is well documented. This case study provides an alternate treatment option for a selected subgroup of patients with areas of progressive periprosthetic osteolysis with a 100% survivorship rate at an average of 5.1 ± 2.4 (range, 1.0 to 10.0) years.

The clinical use of demineralized bone matrix and cancellous bone chips is well supported in the literature [27, 28]. Although commercial preparations vary, these products deliver the necessary osteoconductive and osteoinductive components of bone to the surgical site. Bone grafting has shown success as a treatment method in both retroacetabular osteolysis in THR and periprosthetic osteolysis in TKR [10, 12, 13, 15, 16]. In the current study, treatment of osteolytic lesions included curettage and subsequent packing with bone graft material, effectively resolving the lytic progression.

Several aspects of the current study limit the ability to generalize these results. Adhering to our pre- and intraoperative surgical decision models, the number of cases available for inclusion was limited. Based on our favorable outcomes in this small population, continued use and investigation of this treatment method is justified. While a single surgeon's patient data eliminated variation due to surgical technique, it is recognized that reporting results from one experienced surgeon may not represent outcomes from more widespread use of this technique. This method for treating progressive periprosthetic osteolysis in cementless TKR is primarily dependent on having well-fixed components at the time of revision, which in our study was enhanced through use of bone slurry at index TKR. Its effectiveness for other TKR designs or cemented TKR is unknown.

Fully incorporated grafts occurred in all ten cases in this study, including 7 large defects. These results are similar to other published results for insert exchange in TKR, ranging from 84.6% to 97% complete or near complete graft incorporation into treated osteolytic lesions [15, 16]. However, considering that radiographs tend to underestimate the degree of osteolysis, it is challenging to assign a clear magnitude of the disease treated [29]. It is recognized that the use of CT or MRI provides some benefit for gaining a three-dimensional perspective of the lytic defect, as recently demonstrated by others [30, 31]. MRI has been shown to be more accurate and sensitive than CT for defect detection in the femur, while CT performs with better accuracy in the tibia and in defects less than 2 cm^3^ [31].

## 4. Conclusion

This series of 10 TKRs with progressive periprosthetic osteolysis around well-fixed and well-aligned components that were treated with curettage of the osteolytic lesions, bone grafting of the resultant defect, and polyethylene insert exchange demonstrated excellent results at an average of 5 years of followup with no cases requiring rerevision surgery. The senior surgeon (TP) continues to selectively use this approach and recommends incorporating the surgical decision models (Figures 1 and 2) at the time of revision TKR. However, if this approach is to be utilized, the inclusion criteria outlined must be strictly followed.
